# Repeated H_2_O_2_ exposure drives cell cycle progression in an *in vitro* model of ulcerative colitis

**DOI:** 10.1111/jcmm.12150

**Published:** 2013-10-09

**Authors:** Angela Poehlmann, Kathrin Reissig, Peter Schönfeld, Diana Walluscheck, Antje Schinlauer, Roland Hartig, Wiebke Lessel, Thomas Guenther, Andrew Silver, Albert Roessner

**Affiliations:** a Department of Pathology, Otto-von-Guericke UniversityMagdeburg, Germany; b Institute of Biochemistry and Cell Biology, Otto-von-Guericke UniversityMagdeburg, Germany; c Department of Molecular and Clinical Immunology, Otto-von-Guericke UniversityMagdeburg, Germany; d Academic Department of Histopathology, St. Mark’s HospitalHarrow, Middlesex, United Kingdom; e Colorectal Cancer Genetics, Centre for Digestive Diseases, Blizard Institute, Barts and The London School of Medicine and DentistryLondon, United Kingdom

**Keywords:** hydrogen peroxide, ulcerative colitis, DNA damage checkpoint adaptation, cell cycle progression

## Abstract

The production of hydrogen peroxide (H_2_O_2_) drives tumourigenesis in ulcerative colitis (UC). Recently, we showed that H_2_O_2_ activates DNA damage checkpoints in human colonic epithelial cells (HCEC) through c-Jun *N*-terminal Kinases (JNK) that induces p21^WAF1^. Moreover, caspases circumvented the G1/S and intra-S checkpoints, and cells accumulated in G2/M. The latter observation raised the question of whether repeated H_2_O_2_ exposures alter JNK activation, thereby promoting a direct passage of cells from G2/M arrest to driven cell cycle progression. Here, we report that increased proliferation of repeatedly H_2_O_2_-exposed HCEC cells (C-cell cultures) was associated with (*i*) increased phospho-p46 JNK, (*ii*) decreased total JNK and phospho-p54 JNK and (*iii*) p21^WAF1^ down-regulation. Altered JNK activation and p21^WAF1^ down-regulation were accompanied by defects in maintaining G2/M and mitotic spindle checkpoints through adaptation, as well as by apoptosis resistance following H_2_O_2_ exposure. This may cause increased proliferation of C-cell cultures, a defining initiating feature in the inflammation-carcinoma pathway in UC. We further suggest that dysregulated JNK activation is attributed to a non-apoptotic function of caspases, causing checkpoint adaptation in C-cell cultures. Additionally, loss of cell-contact inhibition and the overcoming of senescence, hallmarks of cancer, contributed to increased proliferation. Furthermore, there was evidence that p54 JNK inactivation is responsible for loss of cell-contact inhibition. We present a cellular model of UC and suggest a sinusoidal pattern of proliferation, which is triggered by H_2_O_2_-induced reactive oxygen species generation, involving an interplay between JNK activation/inactivation, p21^WAF1^, c-Fos, c-Jun/phospho-c-Jun, ATF2/phospho-ATF2, β-catenin/TCF4-signalling, c-Myc, CDK6 and Cyclin D2, leading to driven cell cycle progression.

## Introduction

Ulcerative colitis (UC) is an inflammatory bowel disease (IBD) characterized by periods of inflammatory recurrence and remission, events accompanied by cell death and regeneration of the colonic mucosa. These repeated periods of damage and repair enhance the risk of neoplastic transformation within the cells of the intestinal epithelium 1. Accordingly, the pathogenesis of colitis-associated colorectal cancer (CAC) is attributed to oxidative stress 2, and the generation of reactive oxygen species (ROS) is considered a consequence of inflammation, a hallmark of cancer as proposed by Hanahan and Weinberg 3. Superoxide, hydrogen peroxide and the hydroxyl radical were recognized to play a crucial role in the progression to CAC 4. It is unclear whether inflammation alone is able to induce tumour initiation, although it is generally accepted that chronic inflammation increases cancer risk, and that inflammation is a tumour promoter 5. Novel studies support the first scenario by assuming oxidatively damaged DNA as an initial event 6,7.

**Figure 1:**
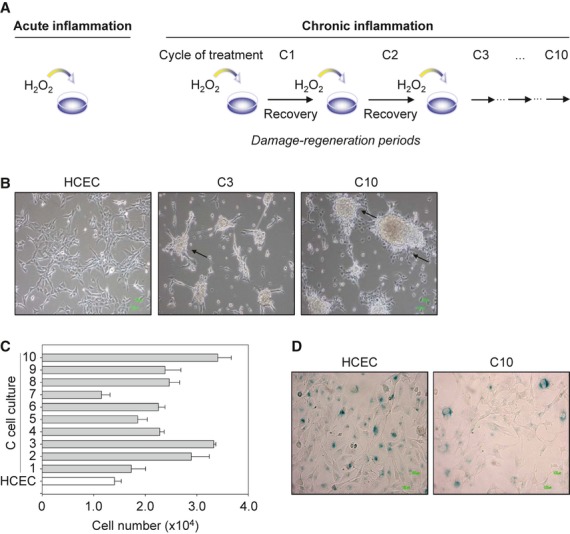
An *in vitro* model of ulcerative colitis showing loss of cell-contact inhibition, increased proliferation, and overcoming of senescence. (A) Study designed to mimic acute and chronic inflammation *via* ROS using H_2_O_2_. The ROS exposure in acute inflammation was mimicked by single H_2_O_2_ treatment. As chronic UC is characterized by damage-regeneration periods, chronic inflammation was simulated by repetitive injury, exposing human colonic epithelial cells (HCEC) to repeated H_2_O_2_ treatment cycles (C)1-C10 with recovery phases in between. In this way, 10 H_2_O_2_-exposed cell cultures were generated and named C-cell cultures C1-C10. C1-C3 cells were generated in the first study 9 and C4-C10 cells in this study. (B) Loss of cell-contact inhibition occurred in C3 cells and continued until C10 cells. Phase contrast micrographs are shown after 5 days of recovery, and arrows indicate loss of cell-contact inhibition, piling up, and thus foci formation. (C) Increased proliferation of C-cell cultures. C1-C10 cells and HCEC cells were cultivated, and cell numbers were counted after 7 days. Data indicate mean ± SD and were obtained from four individual measurements. (D) Cells were grown for 48 hrs, fixed and subsequently stained for ß-galactosidase activity (blue areas).

In a recent study, we simulated inflammation-associated oxidative stress of the epithelium in UC by exposing non-tumour human colonic epithelial cells (HCEC) to periods of three H_2_O_2_ exposures, each followed by periods of cellular recovery (Fig. 1A) 9, generating HCEC cycles (C)1 to C3 (C-cell cultures). Both undetected DNA damage and increased proliferation were found in C1–C3 cells, features that are associated with neoplastic transformation 3. Survival was explained by JNK-dependent cell cycle arrests with caspases, p21^WAF1^ and γ-H2AX identified as the key JNK-regulated proteins. Overexpression of upstream phospho-JNK has been observed in active UC, which further indicates the importance of this pathway *in vivo*. Up-regulation of caspases 3, 8 and 9 was linked to survival and not, as might be expected, to apoptosis 9: caspases guided cells through the G1 and S phase by overriding the G1/S and intra-S checkpoints despite the presence of DNA damage. This non-apoptotic function of the caspases led to the accumulation of cells in the G2/M phase and decreased apoptosis. Survival of oxidatively damaged HCEC cells occurred *via* caspase-mediated γ-H2AX suppression through proteolytic degradation of the DNA damage checkpoint protein ATM, which is upstream of γ-H2AX 9.

Proteins controlling the cell cycle ultimately determine cell fate, such as cell cycle arrest, following DNA damage. Currently, little is known about cell cycle arrest and its precise mechanism and function in the development of UC and UC-associated neoplasia 10. Furthermore, it is not clear whether arrest links reparative with uncontrolled proliferative response. Driven cell cycle progression in the dextran sulphate sodium (DSS)-induced colitis mouse model and in UC patients might be a consequence of a previous cell cycle arrest 11–12. Normally, when DNA damage occurs, DNA damage checkpoints halt the passage of cells through the cell cycle 13,14. In contrast, cells with impaired cell cycle control have selective growth advantages. Thereby, defective maintenance of cell cycle arrest through checkpoint adaptation may cause increased proliferation 16.

JNK is involved in both the acute inflammatory response 17 and the activation of DNA damage checkpoints leading to cell cycle arrest 9. The JNK family consists of two isoforms, JNK1 and JNK2, which are ubiquitously expressed, and of tissue-specific JNK3, all of which have two splicing variants (p54 and p46) 18,19. In many cases, the *Jnk1* gene encodes the p46 protein product, and the *Jnk2* gene encodes the p54 protein product 21. JNK mediates cellular survival and apoptosis, while the cell fate is dependent on the stimuli and the cell type involved 22. However, JNK may only exert a prosurvival function in p53-inactivated cells 23. In the development of UC, the inactivation of the p53 protein is an important early step 24. Thus, the functional disruption of the p53 protein in HCEC cells by its inactivation with the large T-antigen of the SV40 virus 25 enables the correlation of JNK with cellular survival following oxidative stress.

Here, we hypothesize that cells surviving multiple H_2_O_2_ exposures directly pass over from cell cycle arrest to driven cell cycle progression, and that JNK plays a pivotal role in this process. Thereby, dysregulation of JNK seems to switch the signalling pathways from arrest to increased proliferation. In support of our first study 9, the non-apoptotic function of caspases appears to initiate the neoplastic features as they suppress JNK activation and thus JNK-dependent DNA damage checkpoints. The cellular model presented here provides a unique *in vitro* system to investigate the molecular mechanisms that may underlie the early tumourigenic events in CAC, such as driven cell cycle progression. Summing up, this model further supports that chronic inflammation-associated oxidative stress is likely to trigger tumourigenesis.

## Material and methods

### Cell culture

Human colonic epithelial cells, generated by Nestec Ltd (Nestlé Research Center Lausanne, Switzerland 25), were obtained from Professor Pablo Steinberg (Institute of Food Toxicology and Analytical Chemistry, University of Veterinary Medicine Hanover, Germany 26) and were cultured as described previously 9.

### Generation of C-cell cultures C4 to C10

The generation of H_2_O_2_-exposed HCEC cycles (C)1 to C3 has recently been reported by us 9. For the generation of C4-C10 cells, 1 × 10^6^ cells of C3 were seeded into a Petri dish and treated with 200 μM H_2_O_2_ 9. After 24 hrs, the medium was removed, cells were washed twice with PBS, and surviving cells were cultivated until recovery (C4 cells). Then, 1 × 10^6^ cells were seeded into a Petri dish for the next treatment to generate the next C-cell culture. In this way, 10 C-cell cultures (C1-C10 cells) were generated. Untreated HCEC cells were passaged in the same way to serve as controls.

### Inhibition studies

JNK kinase and caspase activities were inhibited by using the JNK inhibitor SP600125 (Enzo, Lörrach, Germany) at a concentration of 50 μM and the pan-caspase-inhibitor Z-VAD-FMK (50 μM, R&D Systems, Minneapolis, MN, USA) as reported earlier 9.

### Immunoblot analysis

One million cells of the respective cell culture were seeded into Petri dishes. Cells were harvested after 48 hrs, and proteins were prepared as described previously 27. The following antibodies were used: JNK, phospho-JNK(Thr183/Tyr185), c-Jun, phospho-c-Jun(Ser63), phospho-c-Jun(Ser73), Cyclin D2, CDK1, CDK2, CDK4, Cyclin B1, c-Fos, phospho-p38(Thr180/Tyr182), phospho-ERK1/2(Thr202/Tyr204), phospo-ATF2(Thr69/71), phospo-ATF2(Thr69), STAT3, phospho-STAT3(Tyr705) (Cell Signaling Technology, Danvers, MA, USA); p21^WAF1^ (Calbiochem, Darmstadt, Germany); β-actin, β–catenin (Sigma-Aldrich, Steinheim, Germany); c-Myc (Abcam, Cambridge, UK); CDK6 (Acris, Antibodies, Herford, Germany); ATF2, TCF4 (Santa Cruz Biotechnology, Santa Cruz, CA, USA); and Sp1 (Novus Biologicals Inc., Littleton, CO, USA). Densitometric analysis of the data was performed by using the GeneTools Software from Syngene (Cambridge, United Kingdom). Fold induction (ratio protein/β-actin) was calculated by using the loading control β-actin.

### ROS assays

Estimation of intracellular ROS: Cells suspended in PBS supplemented with 20 mM glucose were loaded with 2 μM dihydrodichlorofluorescein diacetate (DCFH-DA) for 15 min. at 37°C. Inside the cells, DCFH-DA becomes hydrolysed to DCFH, a probe being oxidized to the fluorescent DCF (excitation at 475 nm and emission at 525 nm) by cellularly formed ROS. Cellular ROS generation was monitored with 2 μg cell protein *per* well at 25°C by using a microplate fluorimeter (Tecan Austria GmbH, Salzburg, Austria). ROS levels were normalized to cellular protein, determined by the BCA Protein assay kit (Pierce, Rockford, IL, USA).

Estimation of extracellular H_2_O_2_: the Amplex red (AR) assay was applied for assessing the H_2_O_2_ concentration in PBS. The nonfluorescent AR becomes oxidized by H_2_O_2_ to the fluorescent resorufin, which was estimated either fluorimetrically (excitation at 560 nm, emission at 590 nm) or photometrically (560 nm) at 37°C. Decomposition of added H_2_O_2_ in HCEC-containing PBS medium was measured photometrically (Fig. 4B). Briefly, aliquots of the PBS medium (with or without H_2_O_2_, or cells or either) were withdrawn at distinct time intervals, and the decline of concentration of added H_2_O_2_ (200 μM) was measured. The assay medium contained 5 μM Amplex red plus horseradish peroxidase (2 units/ml). In addition, as HCEC cells release low amounts of H_2_O_2_ into the surrounding medium, the released H_2_O_2_ was followed fluorimetrically (Fig. 4D). For this purpose, wells of a microplate were supplied with PBS medium supplemented with 5 μM Amplex red plus horseradish peroxidase (2 units/ml) and 2 μg cell protein.

### Proliferation assay, cytokine assay and β-galactosidase staining

Proliferation of HCEC and C4-C10 cells, Il-6 release and the β-galactosidase assay for cellular senescence were performed as described previously 9–28.

### Real-time PCR

cDNA synthesis and PCR were performed as described by us 27.

### Immunohistochemistry

The Department of Pathology, Otto-von-Guericke University, Magdeburg, Germany, provided us with biopsies of intestinal mucosa taken from UC patients. The specimens used were collected from the terminal *ileum*, *caecum*, colon *ascendens*, colon *transversum*, colon *descendens*, colon *sigmoideum* and *rectum*. The mucosal biopsy specimens were formalin-fixed, paraffin-embedded and cut into 2 μm thick sections. The sections were incubated with affinity-purified rabbit monoclonal antibody against p21^WAF1^ (Clone EP147, Epitomics, Burlingame, CA, USA) diluted 1:20 for 32 min at room temperature. The reactions were visualized by DAB detection (iVIEW DAB Detection Kit, VENTANA, Oro Valley, AZ, USA). The slides were counterstained with haematoxylin and cover-slipped in mounting medium.

## Results

### Increased proliferation of C1-C10 cells

Recently, we reported a cellular model of H_2_O_2_-associated colitis (Fig. 1A, 9), showing increased proliferation of C1-C3 cells. To study driven cell cycle progression in more detail and to investigate the underlying molecular mechanisms, we extended the exposure of cells to H_2_O_2_, followed by a period of recovery up to a 10th treatment cycle (C10 cells; Fig. 1A). The newly generated H_2_O_2_-exposed HCEC cell cultures were denoted as C4-C10 cells.

First, we considered the morphological phenotype of C-cell cultures. After application of only three H_2_O_2_ treatments, the cells lost cell-contact inhibition and piled up to form foci (Fig. 1B), a characteristic feature of transformed cells 29–30. As loss of cell-contact inhibition has been attributed to cell expansion, we then determined the proliferation capacity of C4-C10 cells. C4-C10 cells showed increased proliferation after 7 days except for C7 cells (Fig. 1C). To examine whether increased proliferation results from overcoming senescence, the β-galactosidase activity assay was applied to C10 and HCEC cells. A decreased β-galactosidase-dependent activity staining of C10 cells was found (Fig. 1D), which clearly indicates an overcoming of senescence. Hence, loss of cell-contact inhibition and overcoming of senescence may contribute to the increased proliferation observed in C-cell cultures.

### Repeated H_2_O_2_ exposure selectively decreases JNK activation and down-regulates p21^WAF1^

**Figure 2:**
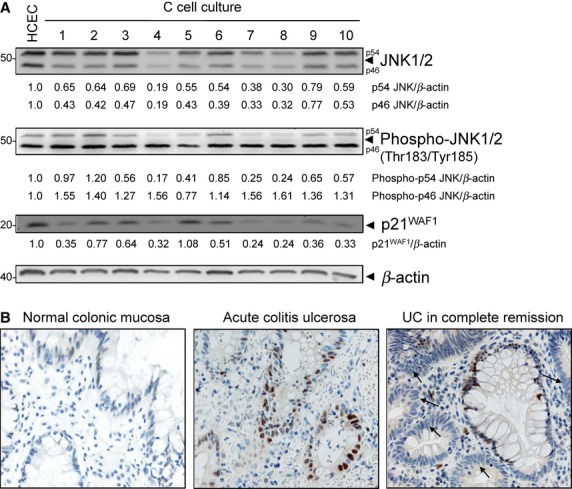
Altered JNK activation is associated with p21^WAF1^ down-regulation in C-cell cultures. (A) Lysates from C-cell cultures and human colonic epithelial cells (HCEC) were immunoblotted with anti-JNK, -phospho-JNK, -p21^WAF1^ and -β-actin antibodies. β-actin served as loading control, and fold expression relative to HCEC cells is given below the blots. P21^WAF1^ immunoblotting of HCEC and C1-C3 cells is published in 9. (B) Immunohistochemical analysis of p21^WAF1^ in normal colonic mucosa, in active UC and in UC in complete remission. Arrows indicate proliferative cells with marginal or no expression.

We have recently shown that H_2_O_2_ activates DNA damage checkpoints through JNK 9. Consequently, the expression of activated, phosphorylated JNK (phospho-JNK) was analysed in C-cell cultures and compared with that of HCEC cells. Densitometric analysis revealed the down-regulation of phospho-p54 JNK starting from C3 cells (Fig. 2A, Fig. S1). Importantly, the phospho-p46 JNK was increased relative to HCEC except for C5 cells (Fig. 2A, Fig. S1). These results, taken together, demonstrated an increased activation of p46 JNK, but a decreased activation of p54 JNK. In addition, the down-regulation of total JNK (p46 and p54) was observed along all C-cell cultures (Fig. 2A).

In line with an increased proliferation of C-cell cultures and with altered JNK activation, repeated H_2_O_2_ exposures initiated down-regulation of p21^WAF1^, except for C5 cells (Fig. 2A). Similarly, we recently reported down-regulation of p21^WAF1^ for C1-C3 cells 9, and this down-regulation can now be confirmed until C10 cells. Both altered JNK activation and p21^WAF1^ down-regulation might drive cell cycle progression, while both changes follow a previous JNK-dependent cell cycle arrest *via* p21^WAF1^ 9. This indicates an important function of cell cycle arrest and especially of p21^WAF1^ in UC. In support, we were able to detect only marginal or no expression of p21^WAF1^ in proliferative cells in samples from UC patients in complete remission as compared with basal expression in normal colonic mucosa and p21^WAF1^ overexpression in biopsies from patients with acute UC (Fig. 2B).

### JNK inactivation and p21^WAF1^ down-regulation act as pathogenetic factors

**Figure 3:**
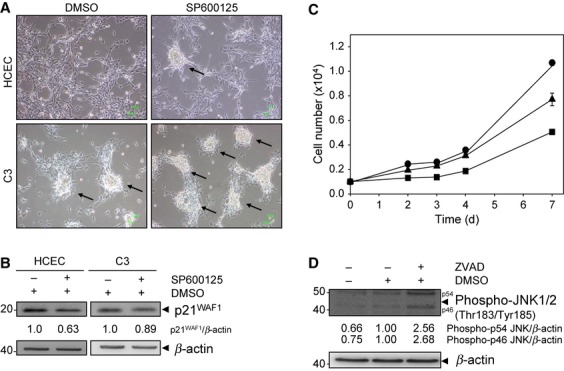
JNK inactivation and p21^WAF1^ down-regulation as pathogenetic factors. (A) Phase contrast micrographs of human colonic epithelial cells (HCEC) and C3 cells 48 hrs after treatment with DMSO or 50 μM SP600125. Arrows indicate loss of cell-contact inhibition, piling up, and foci formation. (B) Lysates from HCEC and C3 cells 24 hrs after treatment with DMSO or 50 μM SP600125 were immunoblotted with anti-p21^WAF1^. β-actin served as loading control, and fold expression relative to HCEC cells is given below the blots. (C) Cell numbers of HCEC cells treated with DMSO (▴), SP600125 (▪), and without treatment (•) after 2, 3, 4 and 7 days are shown. The data represent mean ± SD of four individual measurements. (D) Lysates from C3 cells 72 hrs after treatment with DMSO or 50 μM of the pan-caspase-inhibitor Z-VAD-FMK were immunoblotted with anti-phospho-JNK. β-actin served as loading control, and fold expression relative to HCEC cells is given below the blots.

To examine a possible relationship between decreased JNK activation and the down-regulation of p21^WAF1^, we used C3 cells in which selectively decreased JNK activation appears to have established, as well as HCEC cells for JNK inhibition studies. First, we treated both cell cultures with SP600125, a reversible ATP-competitive JNK inhibitor, and analysed cell morphology. Inhibition of overall JNK activity attenuated cell-contact inhibition in C3 cells and, most notably, induced loss of cell-contact inhibition in HCEC cells (Fig. 3A). This finding supports the hypothesis that JNK inactivation induces loss of cell-contact inhibition, triggering HCEC cell transformation. As JNK inactivation was restricted to the p54 splicing variants in C-cell cultures, we hypothesize that selective p54 JNK inactivation triggers HCEC cell transformation as shown by the loss of cell-contact inhibition (Fig. 1B). Second, we proved a direct linkage between JNK inactivation and p21^WAF1^ down-regulation. Indeed, we found p21^WAF1^ down-regulation in HCEC and C3 cells following inhibition of JNK activity (Fig. 3B). Hence, p21^WAF1^ is a JNK-regulated protein in HCEC and C3 cells. Accordingly, we suggest a relationship between decreased JNK activation and down-regulation of p21^WAF1^ as a general mechanism for C-cell cultures. In support of the observed permanent JNK inactivation starting from C3 cells (Fig. 2A), transient inhibition of activity of both JNK splicing variants by using SP600125 in HCEC cells was not sufficient to induce increased proliferation (Fig. 3C). Thus, we suggest that selective JNK inactivation is required, which has to be established permanently and not transiently. Moreover, we cannot exclude that selective JNK activation may be needed for oncogene activation, or that an interplay between JNK activation and inactivation is important for driven cell cycle progression. In summary, there is good reason to postulate that JNK inactivation accompanied by p21^WAF1^ down-regulation acts as pathogenetic factor that induces loss of cell-contact inhibition.

### Caspases suppress JNK activation in C3 cells

In our previous study, it was shown that caspases 3, 8 and 9 drive progression through the cell cycle in C1-C3 cells as a consequence of oxidative stress 9. This is facilitated by progression of cells through the G1 and S phase following circumvention of DNA damage checkpoint control. In addition, we have demonstrated a caspase-dependent activation of JNK following oxidative stress and its suppression following recovery from oxidative stress 9. We now show that caspase inhibition induced the up-regulation of phospho-p46 JNK and, most notably, of phospho-p54 JNK in C3 cells (Fig. 3D). Thus, the down-regulation of phospho-p54 JNK was mediated through caspase activity. This impairment appears to switch the cellular signalling pathways from cell cycle arrest to an increased proliferation, which could be a general feature of C-cell cultures. Also, the inhibition of caspase activity in C3 cells led to up-regulation of p21^WAF1^ 9, which further supports our hypothesized link between p21^WAF1^ down-regulation and decreased JNK activation. These data also suggest that caspases pushed cells over the checkpoints *via* suppression of JNK activation. However, the functions of caspases observed in our cellular model seem to be restricted to their activities rather than to their expression levels as down-regulation of caspase 3, 8 and 9 was detected (Fig. S2).

### Exogenous H_2_O_2_ induces intracellular ROS generation in C5 and C10 cell cultures

**Figure 4:**
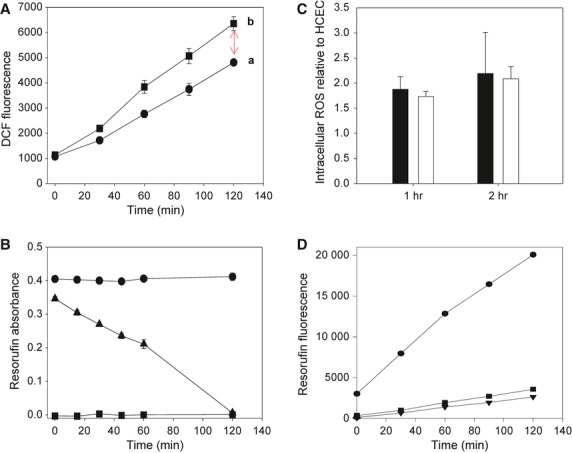
H_2_O_2_-induced reactive oxygen species (ROS) generation in human colonic epithelial cells (HCEC). The ROS generation by HCEC, C5 and C10 cells, measured either with DCFH or Amplex Red, is shown. (A) Intracellular ROS generation by HCEC cells (•) and by HCEC cells treated with 200 μM H_2_O_2_ (•). The arrow indicates H_2_O_2_-induced ROS generation (oxidative stress). The data represent mean ± SD of twelve individual measurements. (B) Time course of the concentration of H_2_O_2_ in PBS medium without (•) and with HCEC cells (10 μg protein of ml; ▴). For comparison, the release of H_2_O_2_ into the medium is also shown (•). The data represent mean ± SD of three individual measurements. (C) X-fold increase in intracellular ROS relative to HCEC cells is shown for C5 (•) and C10 (□) cells. The data represent mean ± SD of 12 individual measurements. (D) Time course of the release of H_2_O_2_ from HCEC (•), C5 (•) and C10 cells (▪). The data represent mean ▾ SD of 12 individual measurements.

Intracellular elevated ROS levels are known to play a crucial role in cell proliferation 31 and tumourigenesis 32. Hence, we investigated intracellular ROS generation in C-cell cultures. First, the intracellular ROS generation was estimated in HCEC cells and in HCEC cells subjected to a single H_2_O_2_ exposure (Fig. 4A). We found that (*i*) HCEC cells generate intracellular ROS even without exposure to exogenous H_2_O_2_ (Fig. 4A, trace a); and that (*ii*) H_2_O_2_-treated HCEC cells have a higher ROS generation (Fig. 4A, trace b). This higher ROS generation may be partly contributed to the diffusion of added H_2_O_2_ across the plasma membrane of HCEC cells. As H_2_O_2_ added to the HCEC cell suspension decomposed completely within 2 hrs (Fig. 4B), there is good reason to speculate that after this incubation period, the increased ROS generation (Fig. 4A, trace b) results from the treated HCEC-cell culture. ROS generation in the C5 and C10 cells was then measured in comparison with HCEC cells (Fig. 4C). An increase in ROS levels was found in the C5 and C10 cells compared with HCEC cells. These data support the hypothesis that exogenous H_2_O_2_ further stimulated intracellular ROS generation. However, the question arises if cells release H_2_O_2_ into the media. Untreated HCEC cells rapidly release H_2_O_2_ into the media (Fig. 4D). In contrast, C5 and C10 cells release H_2_O_2_ into the media, but to a much lesser extent. This process might be associated with increased intracellular ROS production, promoting proliferation of these and probably proliferation of other C-cell cultures as well.

### Expression of oncogenic transcription factors is associated with driven cell cycle progression in C-cell cultures

**Figure 5:**
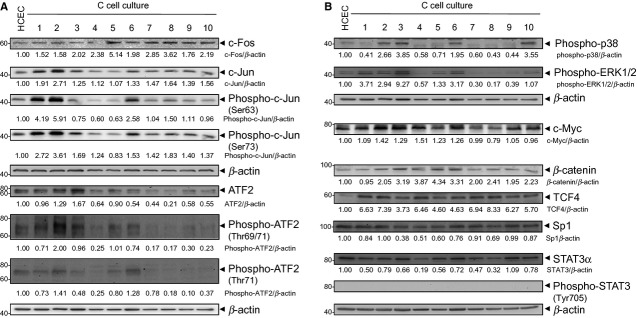
Involvement of oncogenic transcription factors in the H_2_O_2_-associated colitis model. (A) Lysates from C1-C10 cells and human colonic epithelial cells (HCEC) were immunoblotted with anti-c-Fos, -c-Jun, -phospho-c-Jun, -ATF2, -phospho-ATF2 and -β-actin antibodies. β-actin served as loading control, and fold expression relative to HCEC cells is given below the blots. c-Fos immunoblotting of HCEC and C1-C3 cells is published in 9. (B) Lysates from C1-C10 cells and HCEC cells were immunoblotted with anti-phospho-p38, -phospho-ERK1/2, -c-Myc, -β-catenin, -TCF4, -Sp1, -STAT3, -phospho-STAT3, and -β-actin antibodies. β-actin served as loading control, and fold expression relative to HCEC cells is given below the blots. c-Myc and β-catenin immunoblotting of HCEC and C1-C3 cells is published in 9.

Next, we analysed the expression of oncogenic transcription factors that possibly drive cell cycle progression. First, we focused on the transcription factors that constitute AP-1 components, such as c-Fos, c-Jun and ATF2 (Fig. 5A). Second, we analysed transcription factors that are non-AP-1 components, such as c-Myc, Sp1, β-catenin/TCF4 and STAT3 (Fig. 5B).

c-Fos was overexpressed in C1-C10 cells, with the highest expression levels detected in C5 and C8 cells (Fig. 5A). Importantly, the expression of c-Jun was prolonged, being highest in the C1, C2, C8 and C10 cells, and that of phospho-c-Jun(Ser63) was highest in the C1, C2, C6 and C8 cells (Fig. 5A). However, Ser63 was phosphorylated to a higher extent than Ser73, but the expression of phospho-c-Jun(Ser73) was prolonged apart from C5 cells (Fig. 5A). ATF2 was mostly expressed in C3 cells and phospho-ATF2(Thr69/71) in C2 cells, such as phospho-c-Jun (Fig. 5A). It is worth noticing that up-regulation of phospho-ATF2(Thr71) was less, suggesting that ATF2 phosphorylation may be mainly ascribed to Thr69. Taken together, up-regulation of the phosphorylated AP-1 components c-Jun and ATF2 occurred in C-cell cultures and presumably serves as an initial molecular proliferation-driving event. As phospho-p54 JNK is down-regulated in C-cell cultures, phosphorylation of the AP-1 components seems to be mediated by phospho-p46 JNK. We also observed up-regulation of activated p38 in C2, C3, C6 and C10 cells, and also of activated ERK1/2 in C1, C2, C3, C5 and C6 cells (Fig. 5B), assuming their potential involvement in AP-1 phosphorylation 33.

In the case of non-AP-1 transcription factors, slight up-regulation of c-Myc with peaks in C2 and C4 cells was found (Fig. 5B). Immunoblotting of C-cell cultures further revealed that TCF4 expression was increased along all C-cell cultures, and β-catenin levels were elevated in C2-C10 cells (Fig. 5B). The aforementioned increased β-catenin/TCF4 may induce c-Myc and c-Jun gene expression 34–35. In addition, down-regulation of Sp1 and STAT3 was detected (Fig. 5B), suggesting that low levels of both proteins drive cell cycle progression of C-cell cultures. Importantly, we observed elevated levels of proliferation-stimulating Il-6 in C3 and C10 cells (Fig. S3).

### Cyclin D2 and CDK6 overexpression drives cell cycle progression

**Figure 6:**
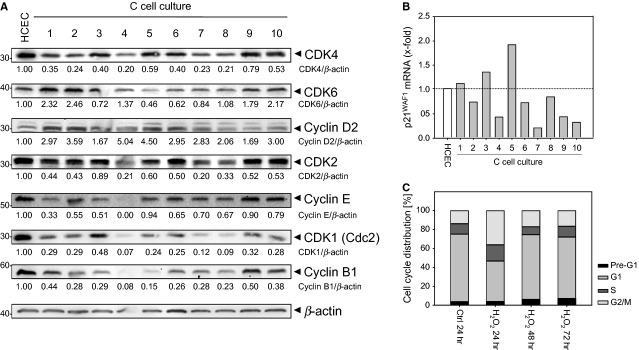
Driven cell cycle progression, associated with overexpression of CDK6 and Cyclin D2, is caused by checkpoint adaptation and apoptosis resistance. (A) Lysates from C1-C10 cells and human colonic epithelial cells (HCEC) were immunoblotted with anti-CDK4, -CDK6, -Cyclin D2, -CDK2, -Cyclin E, -CDK1, -Cyclin B1, and -β-actin antibodies. β-actin served as loading control, and fold expression relative to HCEC cells is given below the blots. (B) RNA from C1-C10 cells and HCEC cells was transcribed into cDNA, and real-time PCR was conducted for p21^WAF1^ mRNA and β2-Microglobulin mRNA expression. (C) H_2_O_2_-treated C10 cells showed S and G2/M arrest after 24 hrs and increased G1 cell population but no apoptosis induction (Pre-G1) after 48 and 72 hrs. The data are representative of three independent experiments.

Next, we analysed the expression of cell cycle regulators involved in different phases of the cell cycle (Fig. 6A). We observed up-regulation of the early G1-specific cell cycle regulators, CDK6 and Cyclin D2, required for G1 progression in C-cell cultures. In contrast, S and G2/M markers, CDK2, Cyclin E, and CDK1 and Cyclin B1, respectively, were down-regulated as well as the G1 marker CDK4. The highest expression of CDK6 was detected in the C1, C2, C4, C9 and C10 cells. Cyclin D2 expression was prolonged, highest in the C1, C2, C4, C5 and C10 cells. Importantly, CDK6 and Cyclin D2 overexpression might facilitate the passage of cells through the G1/S checkpoint and, therefore, might drive cell cycle progression with consequences for tumourigenesis should this occur *in vivo*. We also linked p21^WAF1^ down-regulation to decreased mRNA expression with the exception of C1, C3 and C5 cells (Fig. 6B), which will further reduce the effectiveness of the G1/S, intra-S and G2/M checkpoints.

### Repeated H_2_O_2_ exposure caused checkpoint adaptation

Treatment of C10 cells with H_2_O_2_ resulted in S and G2/M arrest, but led to apoptosis resistance after 24 hrs (Fig. 6C) while apoptosis induction was observed in HCEC cells 9. Instead, arrested C10 cells subsequently re-entered the cell cycle as an increased G1 cell population was observed 48 and 72 hrs after treatment. Thus, C10 cells show a defect in the maintenance of the G2/M cell cycle arrest, and damaged cells enter mitosis because of adaptation of the G2/M and mitotic spindle checkpoints, which led to accumulation of cells in G1, resistant to H_2_O_2_. These molecular events may cause increased proliferation of C-cell cultures. We further suggest that checkpoint-adapted C10 cells were selected by their enhanced viability, and this may consequently contribute to increased proliferation.

## Discussion

In this study, we investigated the question of whether sole inflammation-associated ROS generation drives cell cycle progression of HCEC cells, and whether dysregulated DNA damage checkpoints display the link to unrestricted proliferation, a hallmark of cancer 3. Inflammation-associated ROS generation was mimicked by the exposure of HCEC cells to exogenous H_2_O_2_. We reported previously that an activation of DNA damage checkpoints occurs *via* JNK activation after H_2_O_2_ exposure 9. In the present study, we detected dysregulated JNK activation following repeated H_2_O_2_ exposures, suggesting an ineffective checkpoint control and, therefore, cell cycle progression. Subsequent down-regulation of p21^WAF1^, which is down-stream of JNK, appeared to be the driving force for cell cycle progression. Thus, the decrease in JNK expression, altered JNK activation and p21^WAF1^ down-regulation might cause increased cellular proliferation. Furthermore, with altered JNK activation, activated p38 and ERK1/2 were detected, supporting the observation that these MAPKs, which are known to be involved in survival 36–37, also play a role in our cellular model of UC. Inhibition of total JNK activity impaired cell-contact inhibition in HCEC cells. Thus, loss of cell-contact inhibition observed in C-cell cultures seems to be the result of p54 JNK inactivation, serving as a potential pathogenetic factor.

### Altered JNK activation and p21^WAF1^ down-regulation drive cell cycle progression

A detailed understanding of the role of JNK in tumourigenesis and in the control of the cell cycle is currently not available. However, as already mentioned above, we recently reported the induction of DNA damage checkpoints *via* JNK 9, which supported the discovered link between JNK and intestinal damage in UC 38–39. In the present study, we found driven cell cycle progression in association with the down-regulation of phospho-p54 JNK in C-cell cultures. Moreover, loss of cell contact inhibition in C-cell cultures was attributed to p54 JNK inactivation. In support, JNK suppresses Ras-stimulated transformation of fibroblasts 40. Summing up these observations, we are encouraged to hypothesize that p54 JNK has a tumour suppressor function in our *in vitro* model of UC. In contrast, JNK1, mostly p46 JNK, is known to act as a tumour suppressor in the intestine, but, importantly, tumourigenesis was linked to p21^WAF1^ down-regulation 41, and this down-regulation was also observed in our *in vitro* model. In addition, JNK is activated in most hepatocellular carcinomas 42, and cell proliferation required JNK1-dependent p21^WAF1^ down-regulation 43, presumably because c-Jun is able to negatively regulate p53 transcription, and thus, p21^WAF1^ expression 44. Our investigations showed that JNK mediates p21^WAF1^ expression 9, a process that is probably independent of p53 because of its inactivation through the SV40 virus 25. Thus, p21^WAF1^ down-regulation seems to be the result of a negative regulation of JNK phosphorylation *via* caspase activity. Interestingly, caspases suppress both phospho-p46 JNK and phospho-p54 JNK in C3 cells. However, as total phospho-p46 JNK is up-regulated in C-cell cultures, another molecular mechanism seems to counteract caspase-mediated suppression. Down-regulation of p21^WAF1^ occurred early and was as efficient as p53 mutation, which is in line with the proposed role of p21^WAF1^ as a potential tumour suppressor in the colon 45–49. Here, we show decreased mRNA and protein levels of the JNK-regulated protein p21^WAF1^ in C4 and C6-C10 cells, indicating less transcriptional p21^WAF1^ induction. We presume that p21^WAF1^ down-regulation following altered JNK activation leads to driven progression of cells through cell cycle phases in our cellular model of UC.

Overall, JNK can act as a tumour promoter or suppressor, depending on the cell type. Importantly, in our study, the selective phosphorylation of JNK splicing variants p46 and p54 appeared to play an important role in driven cell cycle progression. Blonska and Lin found selective phosphorylation of p54, but not that of p46 JNK, in lymphocytes activation and proliferation 50. In line with our data, levels of the JNK target c-Jun and of phospho-c-Jun were increased in *Jnk2*^−/−^ fibroblasts, with p54 JNK nearly lost 51. Also, they showed that JNK2 with predominant p54 protein seems to inhibit JNK1 with predominant p46 protein. This could explain phospho-p54 JNK down-regulation with up-regulation of phospho-p46 JNK in our model. Taken together, our study sheds light on how the different activated JNK splicing variants operate. Obviously, these splicing variants appear to be more important than the detection of overall JNK activation.

### Overexpression of oncogenic transcription factors and G1 cell cycle regulators

Expression of immediate early response genes, such as AP-1 components c-Fos, c-Jun and ATF2, has been linked to cellular transformation 52. In line with this, we observed prolonged up-regulation of c-Fos in C-cell cultures. Strong up-regulation of phospho-c-Jun and phospho-ATF2, both JNK-regulated proteins, was found in C1-C3 and C6 cells, and in C2 and C6 cells, respectively. As we observed overall up-regulation of phospho-p46 JNK in C-cell cultures, we propose that it activates c-Jun and ATF2. However, they can also be phosphorylated through ERK and p38 33–53. We detected activation of these MAPKs in the respective C-cell cultures. In addition, Wisdom *et al*. found prolonged expression of unphosphorylated c-Jun, which may stimulate G1 progression as reported for fibroblasts 54. They also found that phosphorylated c-Jun protects cells from UV-induced apoptosis. Both stimulated G1 progression, and apoptosis resistance may also be mediated through c-Jun in our model. In this context, positive regulation of c-Jun expression is probably induced by phosphorylated c-Jun/ATF-2 and c-Fos in C-cell cultures as reported by Angel *et al*. 55. Furthermore, cellular transformation induced by Ras requires c-Jun 56, and c-Jun protects early stages of hepatocellular carcinomas in mice against apoptosis 57. C-Jun mediates its proliferative effects through suppression of tumour suppressors, such as p53 and p21^WAF1^, while triggering positive cell cycle regulators, such as CDK’s and Cyclins 58–59. We propose an important role for the AP-1 components that drive HCEC cell cycle progression. Thus, targeting AP-1 components, such as ATF2, seems to be an efficient therapeutic strategy 60.

In UC, increased expression of the c-Myc proto-oncogene has been linked to cellular proliferative response of inflamed colonic mucosa 49–61. In this study, c-Myc was overexpressed already after the earliest H_2_O_2_ exposures (C1-C6 cells). Interestingly, negative regulation of STAT3 plays a role in balancing the inflammatory milieu 62. In support of this, we detected down-regulation of STAT3 in C-cell cultures. Sp1 is involved in the expression of genes that regulate cell proliferation and tumourigenesis 63, but in our model, we could not detect Sp1 overexpression. Importantly, we noticed increased β-catenin and TCF4 levels in C-cell cultures, suggesting an involvement of the Wnt-pathway 64.

In the context of G1 cell cycle regulators, we observed CDK6 and Cyclin D2 overexpression, suggesting that both enhance G1 phase progression in C-cell cultures. Recently, Cole and colleagues also demonstrated the importance of Cyclin D2 and CDK6 for efficient proliferation and colorectal tumourigenesis following *APC* loss 65.

### Increased proliferation in the extended cellular model of H_2_O_2_-associated colitis

The present study shows an impaired cell-contact inhibition, the overcoming of senescence and increased proliferation of HCEC cells after repetitive H_2_O_2_ exposure. Moreover, we found evidence of H_2_O_2_-induced intrinsic ROS generation in C-cell cultures. Otherwise, C-cell cultures exhibited reduced H_2_O_2_ release into the media, when compared with HCEC cells. Hence, we speculate that added H_2_O_2_ underlies intracellular conversion to other ROS, further stimulating ROS generation and signal transduction, which might be responsible for HCEC cell cycle progression. In addition, mitochondria are able to consume H_2_O_2_ 66, and this consumption increases with higher pH, which is a proliferative trigger 67. In summary, internal ROS generation within C-cell cultures as a consequence of H_2_O_2_ exposure is likely to drive cell cycle progression by operating as an internal carcinogenic trigger, which was also reported by Terzic *et al*. 68. Therefore, the cells are driven through the cell cycle without additional external growth stimulation.

Treatment of JNK-dysregulated C10 cells with H_2_O_2_ revealed a survival mechanism based on a defect in maintaining S and G2/M cell cycle arrests, followed by progression to the next phase, accumulation of cells in G1 and apoptosis resistance. Importantly, although the initiation of G2/M cell cycle arrest occurred earlier, it was shortened and, most notably, could not be maintained, such as the S arrest. As H_2_O_2_-exposed HCEC cells regulate DNA damage *via* JNK-dependent checkpoints 9, we further suggest that adaptation of the G2/M and also of the mitotic spindle checkpoint is caused by altered JNK activation. However, the detailed molecular mechanisms, which might also be important in UC tumourigenesis, should be further investigated.

### Extending the model enabled the investigation of permanent and transient molecular mechanisms

In our first study, we showed increased proliferation of C1-C3 cells 9, while we could confirm increased proliferation also for the present extended model involving C4-C10 cells. We found a trend of molecular events in C1-C3 cells, which were permanently manifested along C4-C10 cells. Additionally, extending the model enabled us to observe transient molecular events that occur after the third H_2_O_2_ exposure and that appear to be important. In this context, the C5 cells appear to show an extraordinary behaviour along all C-cell cultures, namely they display sole exceptions with regard to the expression of DNA damage checkpoint proteins. In detail, we found (*i* ) down-regulation of p21^WAF1^ along all C-cell cultures except for C5 cells and (*ii* ) up-regulation of phospho-p46-JNK and phospho-c-Jun(Ser73) along all C-cell cultures except for C5 cells. This was paralleled by strongest expression of c-Fos and β-catenin. As the levels of p21^WAF1^, c-Jun, phospho-c-Jun(Ser73), ATF2 and phospho-ATF2(Thr71)/(Thr69/71) in C5 cells approximate to the levels of HCEC cells, we suggest that C5 cells might display countermeasure against the ROS-induced changes. Taken together, the second study gave more detailed insights into the underlying mechanisms for driven cell cycle progression, enabling the creation of the following model.

### Proposed model

**Figure 7:**
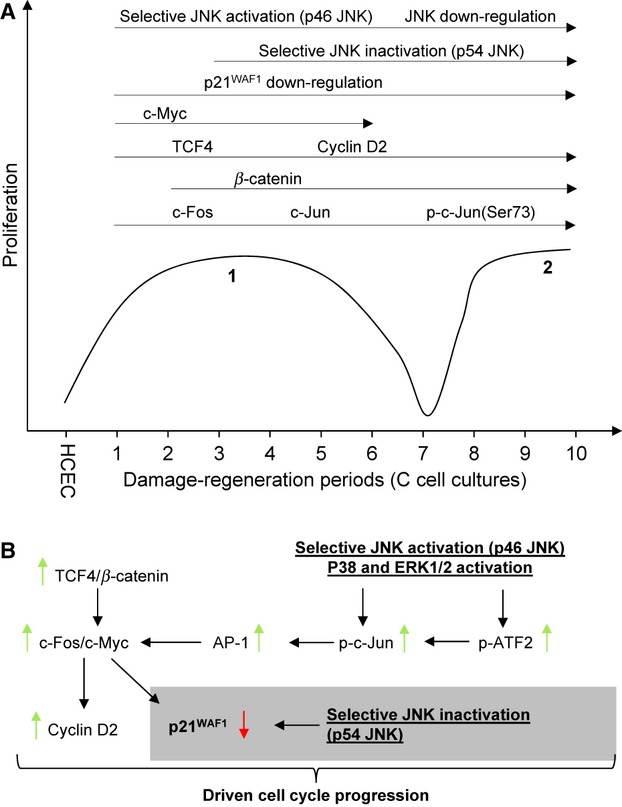
Proposed molecular mechanisms underlying driven cell cycle progression in the *in vitro* model of ulcerative colitis. (A) Increased proliferation showed a sinusoidal pattern consisting of maxima 1 and 2. Permanent molecular events were displayed by an arrow above the proliferation curve. (B) We suggest a model involving an interplay of selective JNK inactivation and p21^WAF1^ down-regulation, selective JNK activation, AP-1 components and β-catenin/TCF4-signalling. Two molecular events may finally lead to p21^WAF1^ down-regulation: selective JNK inactivation and β-catenin/TCF4-dependent suppression of p21^WAF1^.

On the basis of data presented here, we observed a dominant proliferation maxima consisting of C1-C7 cells with a peak in C3 cells (maxima 1) and a beginning second maxima consisting of C8-C10 cells (maxima 2), suggesting a model of sinusoidal proliferation (Fig. 7). (A) We found permanent molecular events along the C-cell cultures, as well as those that appear to be only transient. The following permanent molecular changes were observed in C-cell cultures: (*i* ) total JNK down-regulation, (*ii* ) selective JNK activation (p46), (*iii* ) selective JNK inactivation (p54) starting from C3 cells, (*iv*) p21^WAF1^ down-regulation and (*v*) increased levels of c-Fos, c-Jun, phospho-c-Jun(Ser73), TCF4 and Cyclin D2. β-catenin was up-regulated in C2-C10 cells. Interestingly, c-Myc was up-regulated in C1-C6 cells. In contrast, we noticed transient up-regulation of phospho-c-Jun(Ser63), ATF2, phospho-ATF2(Thr69/71), CDK6, as well as activation of p38 and ERK1/2 in maxima 1. In maxima 2, CDK6, p38 and c-Jun were transiently activated. (B) Taken together, we propose an interplay of selective JNK inactivation, p21^WAF1^ down-regulation, selective JNK activation, p38/ERK1/2 activation, involvement of AP-1 components and β-catenin/TCF4-signalling. Thereby, two molecular pathways may account for p21^WAF1^ down-regulation: (*i* ) selective JNK inactivation (p54) and (*ii* ) c-myc induction. C-myc, in turn, can be activated through (*i* ) *β*-catenin/TCF4 or (*ii* ) selective JNK activation (p46) and/or p38/ERK1/2 activation, inducing AP-1 components c-Jun and ATF2. We propose that early p21^WAF1^ suppression (C1 and C2) is caused by selective JNK activation and/or p38/ERK activation *via* AP-1-dependent c-Myc induction that suppresses p21^WAF1^. Later p21^WAF1^ down-regulation starting from C3 cells appears to be mainly attributed to the interplay of altered JNK activation and β-catenin/TCF4. This also suggests a relationship between the Wnt and JNK pathway. This model is in line with the results of Saadeddin *et al*., who reported such a coordination of both pathways through creation of a transcriptional complex consisting of β-catenin/TCF4 and c-Jun (AP-1), which then activates common target genes, such as c-Myc 34. To the best of our knowledge, we are the first to demonstrate that selective JNK inactivation might provide a link to p21^WAF1^ down-regulation and, therefore, the switch from cell cycle arrest to increased cell cycle progression. Finally, we speculate that all of the molecular events presented in this study might operate in UC tumourigenesis. Moreover, it is conceivable that selected genetic events are responsible for the proliferation bottleneck seen around cycle 7. Future studies will include deep-sequencing of C7- and C10-cell cultures to identify genetic mutations that can then be related to the mutation profiles of cancer arising in the colitic bowel.

## Supporting Information

Figure S1 Down-regulation of p54 splicing variants of phospho-JNK (▾) and up-regulation of p46 splicing variants of phospho-JNK (•) in C-cell cultures. Lysates from C1–C10 cells and from HCEC cells were immunoblotted with anti-phospho-JNK and -βactin antibodies. x-fold expression is relative to HCEC cells and relative to βactin, which was estimated through densitometric analysis.

Figure S2 Expression of caspase 9, 8 and 3 in C-cell cultures. Lysates from C1–C10 cells and HCEC cells were immunoblotted with anticaspase 9, -caspase 8, -caspase 3 and -b-actin antibodies. -βactin served as loading control, and fold expression relative to HCEC is given below the blots. Data of HCEC and of C1–C3 cells are published in [9].

Figure S3 C3 and C10 cells show increased II-6 release compared with HCEC cells.
