# Supplementary figures and images for: Repeated H2O2 exposure drives cell cycle progression in an in vitro model of ulcerative colitis
Source: J Cell Mol Med. 2013 Oct 9;17(12):1619–31. doi: 10.1111/jcmm.12150 (PMC3914643; doi:10.1111/jcmm.12150)

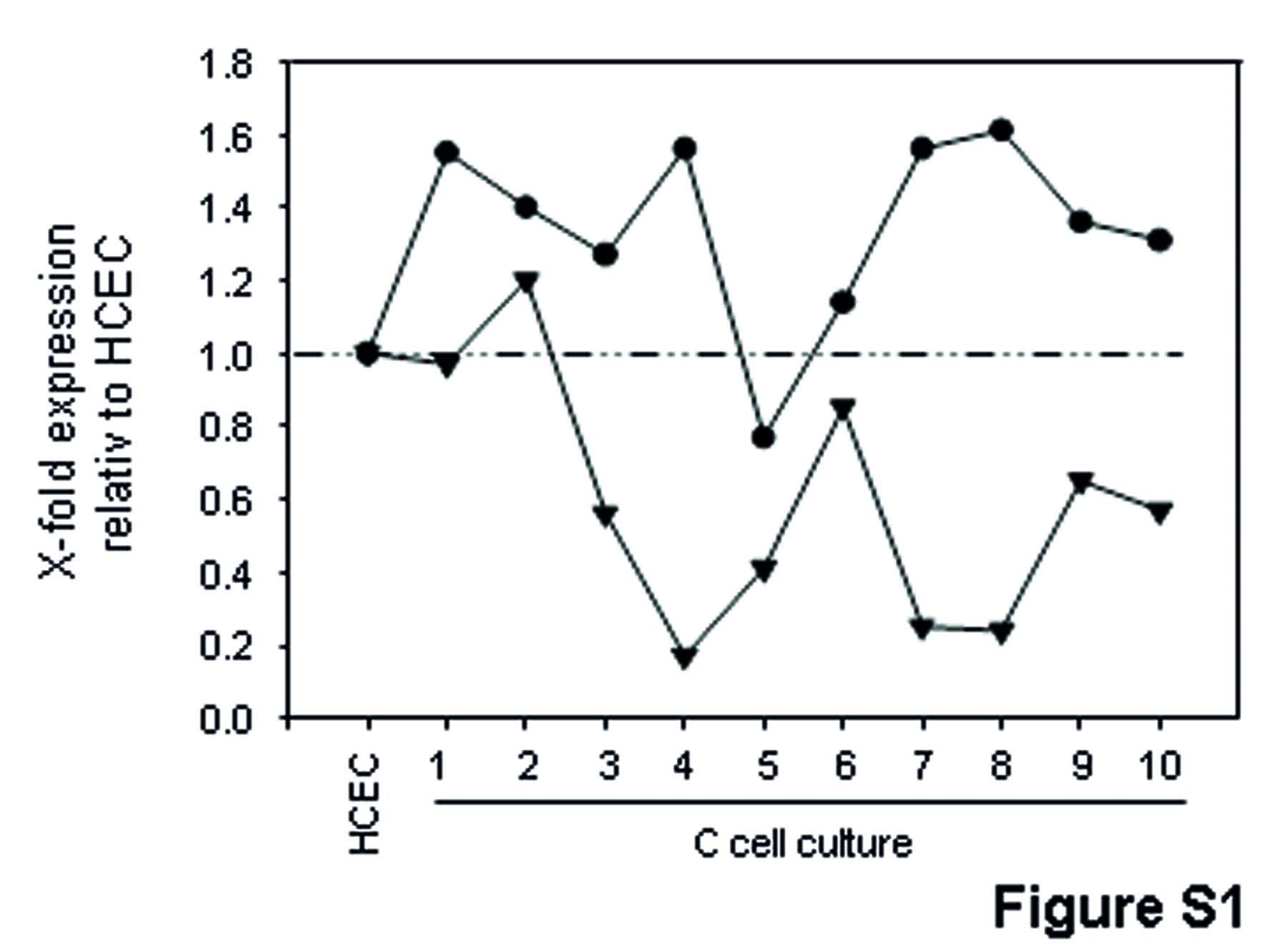

Supplement: Supplementary file 1 [file jcmm0017-1619-sd1.tif]

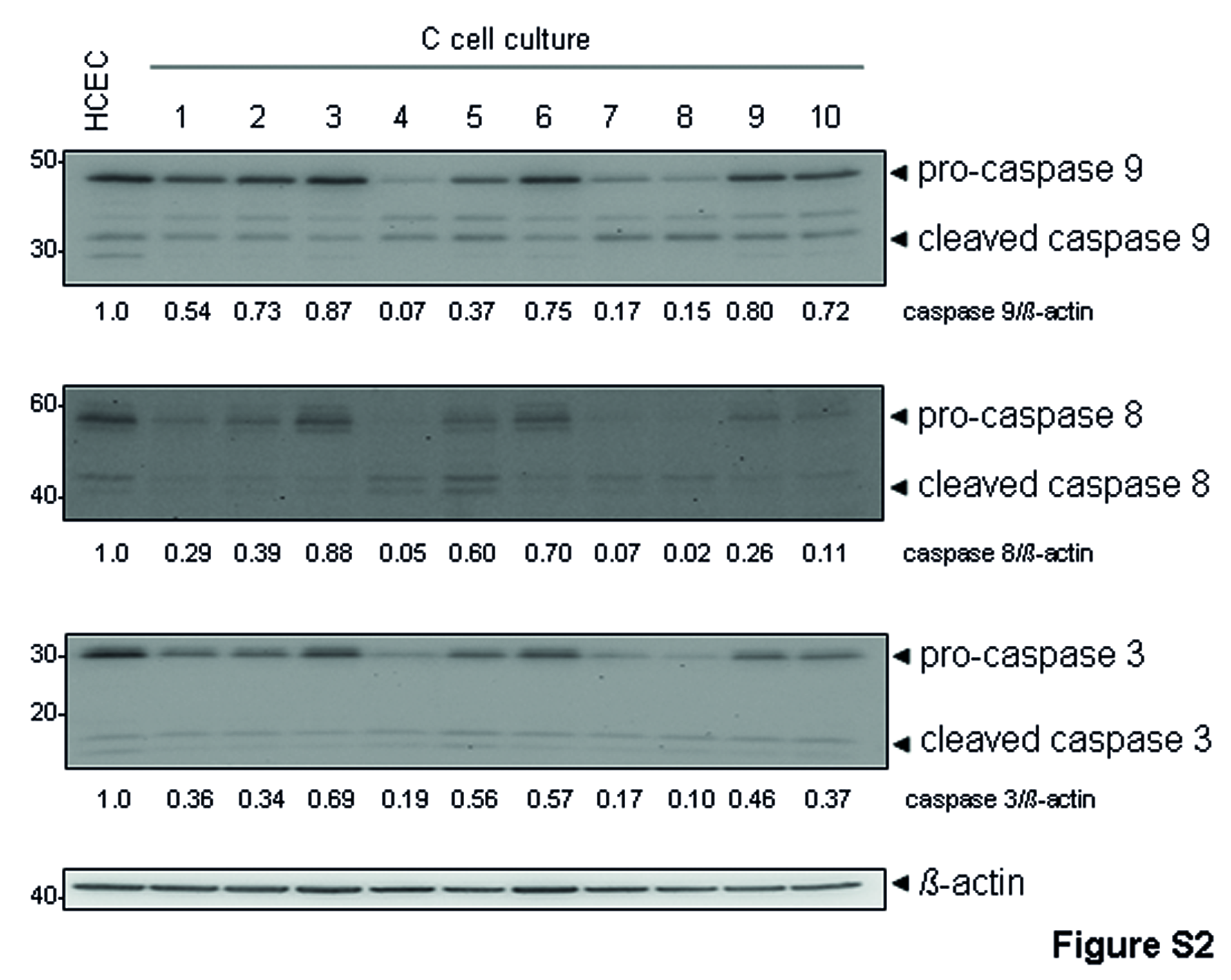

Supplement: Figure S1 — Down-regulation of p54 splicing variants of phospho-JNK (▾) and up-regulation of p46 splicing variants of phospho-JNK (•) in C-cell cultures. Lysates from C1–C10 cells and from HCEC cells were immunoblotted with anti-phospho-JNK and -βactin antibodies. x-fold expression is relative to HCEC cells and relative to βactin, which was estimated through densitometric analysis. [file jcmm0017-1619-sd2.tif]

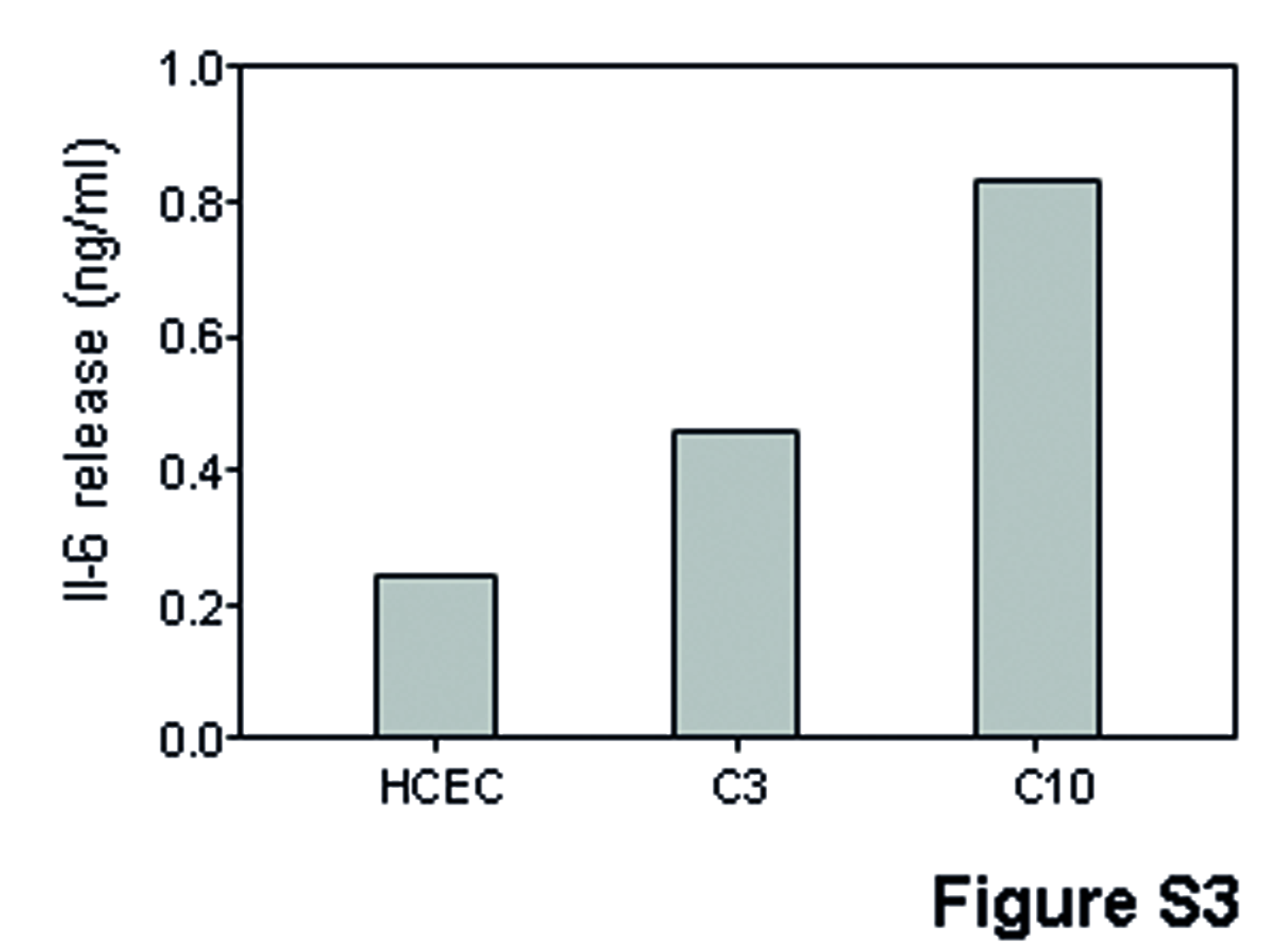

Supplement: Figure S2 — Expression of caspase 9, 8 and 3 in C-cell cultures. Lysates from C1–C10 cells and HCEC cells were immunoblotted with anticaspase 9, -caspase 8, -caspase 3 and -b-actin antibodies. -βactin served as loading control, and fold expression relative to HCEC is given below the blots. Data of HCEC and of C1–C3 cells are published in [9]. [file jcmm0017-1619-sd3.tif]
