# Supplementary material for: Repeated H2O2 exposure drives cell cycle progression in an in vitro model of ulcerative colitis
Source: J Cell Mol Med. 2013 Oct 9;17(12):1619–31. doi: 10.1111/jcmm.12150 (PMC3914643; doi:10.1111/jcmm.12150)
Supplement: Figure S3 — C3 and C10 cells show increased II-6 release compared with HCEC cells. [file jcmm0017-1619-sd4.doc]

**Figure S1** Down-regulation of p54 splicing variants of phospho-JNK (▼) and up-regulation of p46 splicing variants of phospho-JNK (●) in C cell cultures. Lysates from C1−C10 cells and from HCEC cells were immunoblotted with anti-phospho-JNK and -*ß*-actin antibodies. x-fold expression is relative to HCEC cells and relative to *ß*-actin, which was estimated through densitometric analysis.

**Figure S2** Expression of caspase 9, 8 and 3 in C cell cultures. Lysates from C1−C10 cells and HCEC cells were immunoblotted with anti-caspase 9, -caspase 8, -caspase 3 and -*ß*-actin antibodies. *ß*-actin served as loading control, and fold expression relative to HCEC is given below the blots. Data of HCEC and of C1−C3 cells are published in [9].

**Figure S3** C3 and C10 cells show increased Il-6 release compared to HCEC cells.
